# Allergen Micro-Bead Array for IgE Detection: A Feasibility Study Using Allergenic Molecules Tested on a Flexible Multiplex Flow Cytometric Immunoassay

**DOI:** 10.1371/journal.pone.0035697

**Published:** 2012-04-17

**Authors:** Debora Pomponi, Maria Livia Bernardi, Marina Liso, Paola Palazzo, Lisa Tuppo, Chiara Rafaiani, Mario Santoro, Alexis Labrada, Maria Antonietta Ciardiello, Adriano Mari, Enrico Scala

**Affiliations:** 1 Center for Molecular Allergology, IDI-IRCCS, Rome, Italy; 2 Institute of Protein Biochemistry, CNR, Naples, Italy; 3 Allergens Department, Centro Nacional de Biopreparados, Havana, Cuba; King's College London, United Kingdom

## Abstract

**Background:**

Allergies represent the most prevalent non infective diseases worldwide. Approaching IgE-mediated sensitizations improved much by adopting allergenic molecules instead of extracts, and by using the micro-technology for multiplex testing.

**Objective and Methods:**

To provide a proof-of-concept that a flow cytometric bead array is a feasible mean for the detection of specific IgE reactivity to allergenic molecules in a multiplex-like way. A flow cytometry Allergenic Molecule-based micro-bead Array system (ABA) was set by coupling allergenic molecules with commercially available micro-beads. Allergen specific polyclonal and monoclonal antibodies, as well as samples from 167 allergic patients, characterized by means of the ISAC microarray system, were used as means to show the feasibility of the ABA. Three hundred and thirty-six sera were tested for 1 or more of the 16 selected allergens, for a total number of 1,519 tests on each of the two systems.

**Results:**

Successful coupling was initially verified by detecting the binding of rabbit polyclonal IgG, mouse monoclonal, and pooled human IgE toward three allergens, namely nDer s 1, nPen m 1, and nPru p 3. The ABA assay showed to detect IgE to nAct d 1, nAct d 11, rAln g 1, nAmb a 1, nArt v 3, rBet v 1, rCor a 1, nCup a 1, nDer p 1, nDer s 1, rHev b 5, nOle e 1, rPar j 2, nPen m 1, rPhl p 1, and nPru p 3. Results obtained by ABA IgE testing were highly correlated to ISAC testing (r = 0.87, p<0.0001). No unspecific binding was recorded because of high total IgE values.

**Conclusion:**

The ABA assay represents a useful and flexible method for multiplex IgE detection using allergenic molecules. As also shown by our initial experiments with monoclonals and polyclonals, ABA is suitable for detecting other human and non-human immunoglobulins.

## Introduction

There is a growing body of evidence that IgE-mediated allergic diseases have increased over the last 40 years to the point of becoming the most prevalent diseases in the Western world [1], [2]. The diagnostic work-up in allergic diseases is largely based on the use of extracts for both skin tests and *in vitro* IgE testing [3], [4]. A number of pitfalls affect the reliability of these diagnostic approaches, due to the poor quality of the extracts, their unknown component content and sometimes the presence of unwanted contaminants [5].

During the last decades the understanding about allergenic molecules has steadily increased year by year, thus resulting in a remarkable expansion in the identification, characterization and production of allergens, either natural or recombinant (http://www.allergome.org/script/statistic.php).

During the last 10 years, the use of allergenic molecules immobilized on microarrays has been the most fascinating novelty for *in vitro* specific IgE detection [6], allowing at the same time both a higher definition of the patient's IgE sensitization profile and a broader evaluation of IgE reactivity in an unprecedented epidemiological study scale [7]. In fact, besides the use of micro-technology for developing the novel IgE detection method, microarrays like the ISAC (Phadia Multiplexing Diagnostics, PMD, Vienna, Austria) used in the study of Scala et al. [7] bear the intrinsic concept of multiplex testing, where multiplexing means testing a single specimen of the serum sample obtaining hundreds of results at once [8], [9].

A major criticism has been raised toward this multiplexing approach as it is felt to be too rigid in the allergen selection. It seemed to us then that there could be the need for a more flexible but anyhow multiplexed immunoassay platform, allowing the choice of allergens and control molecules (*e.g.* internal assay controls) or for introducing customized target molecules (*e.g.* new allergenic molecules).

A flow cytometric bead array (CBA) was developed to detect soluble factors, like cytokines or mediators, released *in vivo* or *in vitro* during spontaneous or experimentally-induced immune responses [10]–[12]. CBA, in the form of naked fluorescent micro-beads named BD CBA Functional Beads by the producer, can also be customized to detect specific antibodies [10], [11], although not many reports on this specific application are found in the literature [13]. The peculiarity of the BD CBA Functional Beads compared to other micro-bead-based systems [14]–[16] is the use of the flow cytometer, an instrument currently used in almost all large routine and research laboratories.

Drawing inspiration from the early reports of the method developer [10], [11] and the following applications [13], we sought to verify whether allergenic molecules conjugated to the BD CBA Functional Beads could be also useful for specific IgE detection, creating a “flexible” microarray for detecting IgE in human sera. After a series of experiments to set up the BD CBA Functional Beads to meet the need of measuring the less represented immunoglobulin isotype in human serum, we succeeded in detecting specific IgE to allergenic molecules with this new method, named Allergen micro-Bead Array (ABA), obtaining comparable results as from the currently available ISAC microarray assay. The use of the ISAC microarray IgE detection system was decided to be the reference system because of its similar micro-dimension reaction kinetic, and because it is the only multiplexing test using allergenic molecules instead of extracts, bearing all allergenic molecules used in the present study.

## Methods

### Allergen micro-Bead Array - Conjugation Procedure

Allergenic molecules selected for micro-beads conjugation, derived from animal, pollen, and fruit allergenic sources were used. For the first set of experiments, lyophilized purified natural (n) Der s 1, a group 1 mite allergen from *Dermatophagoides siboney*, was provided by Biocen [17] (Havana, Cuba), nPen m 1, a tropomyosin from a shrimp species (*Penaeus monodon*) was purchased from BIAL-Aristegui (Bilbao, Spain), and nPru p 3, a lipid transfer protein (LTP), was purified from peaches (*Prunus persica*) as previously reported [18]. nPru p 3 preparation immobilized on ISAC was not the same used for micro-bead conjugation.

For the second part of the study, using a broader panel of molecules including the three mentioned above, the following additional thirteen allergens were considered: nAct d 1, a cystein protease, and nAct d 11, a ripening-related protein, recently reported as allergen, both purified from green kiwifruit (*Actinidia deliciosa*) [19], [20], nAmb a 1, purified from ragweed pollen (*Ambrosia artemisiifolia*) as reported in literature with minor modifications [21]; nArt v 3, a LTP from mugwort pollen (*Artemisia vulgaris*), purified recombinant (r) Aln g 1, rBet v 1, and rCor a 1, marker allergens from alder pollen (*Alnus glutinosa*), birch pollen (*Betula verrucosa*), hazel tree pollen (*Corylus avellana*), respectively (kindly given by Christian Harwanegg, VBC-Genomics, Vienna, Austria); nCup a 1, the Arizona cypress pollen allergen (*Cupressus arizonica*), nOle e 1, an olive tree pollen allergen (*Olea europaea*), rPar j 2, the parietaria pollen allergen (*Parietaria judaica*), and rPhl p 1, a grass pollen allergen (*Phleum pratense*) (BIAL-Aristegui); nDer p 1, a house dust mite major allergen (*Dermatophagoides pteronyssinus*) (Indoor Biotechnologies, Cardiff, UK); rHev b 5, a marker of natural latex allergy (*Hevea brasiliensis*) (Biomay, Vienna, Austria). Further details on single molecule characteristics are available on the Allergome web site (www.allergome.org) [22]. All allergens were reconstituted with PBS at a concentration of 1 mg/ml and stored at −20°C until used for conjugation.

The color-coded polystyrene micro-beads (Becton Dickinson, New Jersey, USA) of distinct and non-overlapping fluorescence emission intensities [10], [11] were used and covalently coupled with allergenic molecules using the suggested sulfosuccinimidyl 4-N-maleimidomethyl cyclohexane 1-carboxylate (sulfo-SMCC) chemistry. Sulfo-SMCC contains N-hydroxysuccinimide (NHS) ester and maleimide groups. NHS esters react with primary amines at pH 7–9 to form amide bonds, while maleimide groups react with sulfhydryl groups at pH 6.5–7.5 forming stable thioether bonds. The micro-beads herein used colored with a water-insoluble dye, excitable by a 488-nm laser source, with emission at 576 nm (Becton Dickinson) were the same used in other commercially available ready-made CBA assays. The unique difference is that the micro-beads used in the present study were un-conjugated. Thirty different micro-beads having a different mixture of APC-A and APC-Cy7 fluorescence are currently available from the manufacturer [10], [11].

The conjugation procedure consisted of four steps: micro-bead preparation, protein modification, buffer exchange, and protein conjugation.

#### Micro-bead preparation

The BD CBA Functional Beads (Becton Dickinson) were used. Selected micro-beads chosen for conjugation were vortexed vigorously for 30 seconds; 75 µl of micro-beads were taken for 500 test reactions; 1 M 1.9 µl dithiothreitol stored at −20°C (Pierce, Rockford, USA) were added to a micro-centrifuge tube containing the micro-beads and vortexed again for 5 seconds. The mix was incubated on an orbital shaker for 1 h at R.T. in the dark. After incubation, 1 ml of coupling buffer (Becton Dickinson) was added and vortexed for 5 seconds. Afterwards, the micro-beads were centrifuged at 900× *g* for 3 minutes. Afterwards, supernatant removal micro-beads were washed three times and the pellet re-suspended in 20 µl of coupling buffer.

#### Allergenic molecule activation

Ninety µl of selected allergens at a concentration of 1 mg/ml were used for preparing 500 tests. Sulfo-SMCC (Pierce) stock solution (2 mg/ml water solution) was prepared immediately before use, and 2 µl were added to the protein preparation. The mixture was incubated on an orbital shaker for 1 h at R.T. in the dark.

#### Buffer exchange to remove free components

The Bio-Rad Spin Columns (Bio-Rad, Milan, Italy) were washed with coupling buffer twice and then placed in a tube and centrifuged for 2 minutes at 1,000× *g*. After centrifugation, the columns were placed in a new tube and the entire volume of protein/sulfo-SMCC solution was transferred to the spin columns and centrifuged again at 1,000× *g* for 2 minutes.

#### Allergenic molecule conjugation

Activated proteins in the eluate were transferred into the tube containing the previously prepared functional micro-beads and vortexed for 5 seconds. Proteins and micro-beads were incubated for 1 h at R.T. on an orbital shaker in the dark. Two µl of N-ethylmaleimide (2 mg/ml in DMSO, Pierce) were added to the tube containing functional micro-beads and activated protein, vortexed for 5 seconds and incubated for 15 minutes on an orbital shaker. After incubation, 1 ml of the storage buffer was added into the tube and the micro-beads were centrifuged for 3 minutes at 900× *g*. Washes with storage buffer were repeated three times. Afterwards, the micro-bead pellet, re-suspended in 0.5 ml of storage buffer, was stored at +4°C, at a concentration of 6×10^6^ micro-beads/ml.

### Antibodies

#### Monoclonal and polyclonal primary non-human antibodies

To verify the coupling of selected allergens, specific mouse monoclonal (MoAb) and rabbit polyclonal (PoAb) antibodies were used as probes. To detect nDer s 1 onto the micro-beads, two different MoAbs were used, an IgG1 isotype (4E10/E10) and an IgG2b isotype (5F7/H8F2) (Biocen) [17]. For detecting nPen m 1 conjugation to the micro-beads an anti-shrimp tropomyosin PoAb was used (BIAL-Aristegui). nPru p 3 coupling to the micro-beads was assessed using an anti-Pru p 3 MoAb (kindly given by Domingo Barber, Alk-Abelló, Madrid, Spain) [23] and two different anti-Pru p 3 PoAb, the first kindly provided by Alk-Abelló [23] and the second purchased from BIAL-Aristegui. Isotype matched mouse MoAbs to bovine serum albumin and fish parvalbumin and rabbit PoAbs against the same proteins were tested on the three allergen-conjugated beads used for specificity control purposes in preliminary experiments.

#### Human sera

Initially, sera from a total of 167 clinically allergic subjects presenting for a consult at the Center for Molecular Allergology (IDI-IRCCS, Rome, Italy) were selected after testing on the ISAC system, bearing recombinant and natural allergens immobilized in triplicate on a glass slide [9], and following the reported methodology [7]. Samples were selected on the basis of their reactivity to the above three candidate allergens as detected either by the ISAC 89 (VBC-Genomics) or the ISAC 103 microarray systems (PMD). ISAC IgE values are expressed in kU_a_/l. Selected subjects for the Der s 1 ABA testing reported a reliable history of respiratory symptoms of rhinitis and/or asthma suggesting mite allergy and all had IgE positive result using the *Dermatophagoides pteronyssinus* ImmunoCAP test (Phadia, Uppsala, Sweden). A reliable clinical history of food reactions (recent generalized reactions or anaphylaxis) or a positive double blind food challenge test for peach or shrimps, and a positive ImmunoCAP IgE testing for Pru p 3 or Pen a 1, peach LTP and shrimp tropomyosin respectively, were the basis for selecting patients for Pru p 3 and Pen m 1 ABA testing. All subjects were recruited for previously published collaborative studies within our research Center. Allergic control subjects did not report symptoms in relation to exposure to any of the three allergens and had negative allergy in vitro tests.

Human sera to create pools were selected to define the ABA experimental conditions. In order to obtain the highest IgE signal after ABA testing, three distinct pools of sera were set. Sera from twelve patients having nDer s 1 specific IgE on ISAC testing, ranging between 8 and 139 kU_a_/l were mixed together creating the Der s 1 sera pool. The 12 samples were also reactive to other ISAC tested group 1 mite allergens, namely nDer p 1 or nDer f 1. Eighteen nPru p 3 positive patients (ISAC IgE values ranged between 5.4 and 59.29 kU_a_/l), and 21 nPen m 1 positive patients (ISAC IgE values ranging between 5.07 and 92.77 kU_a_/l) were selected for the Pru p 3 and the Pen m 1 sera pools, respectively. In all three cases, although sera were selected on the basis of one of the three specificities - but then used for detecting coupled molecules and ABA optimization - the other two specificities could be present at a lower IgE level, and not avoided on purpose. Three serum samples were selected from patients with one of the three specificities included in our study and not the other two. A forth one had no ISAC detectable nPru p 3 specific IgE. The selection was made in the attempt to mimic the use of discrete antigen specific non-human antibodies, and setting the multiplex approach to verify ABA specificity. Not relevant, but anyway tested allergenic molecules in the same tube, acted as negative controls when the targeted one was tested.

All other human sera used in the study were tested as single non-pooled samples. The number of samples is reported in the Result section for each allergen specificity and detailed in Table S1. Subjects selected for this part of the study had, in case of food allergens, a reliable clinical history or a positive specific food challenge for the related allergenic source, and a reliable clinical history of respiratory symptoms for the remaining allergens.

Total IgE were measured using the ImmunoCAP system (Phadia). As the assay defines IgE concentrations in a range between 1 and 2000 IU/l, sera having very high total IgE levels (>2,000 IU/l) were tested diluted 2 or higher folds in order to get the correct estimation of their total IgE values.

Oral informed consent for blood sampling was obtained from patients or caregivers during the allergy consult as a normal routine procedure during the allergic disease diagnostic work-up. This procedure was approved by our Institutional ethical committee as it is part of a routine procedure where no consent is required by law. Due to the limited amount of serum to be used in the test, no additional blood drawing was necessary. The study was approved by our Institutional ethical committee, at the Istituto Dermopatico dell'Immacolata, Rome, Italy. Informed signed consent was obtained by patients and caregivers when the serum samples were used for the initial part of our study where the new system was set up.

### Multiplex testing procedure

One µl of conjugated micro-beads was used for each test, for each specific allergen. The micro-beads, washed with 0.5 ml of washing buffer as provided by the manufacturer, were centrifuged for 5 minutes at 200× *g*. After removing the supernatant, the micro-beads were re-suspended in capture micro-bead diluent for serum and plasma to a final volume of 50 µl/test, then vortexed and incubated for 15 minutes in the dark at R.T. Fifty µl of micro-bead mix was combined with 50 µl of the biological sample to be tested, being either non-human or human sera, vortexed, and then incubated for 1 hour at R.T. in the dark. PoAbs, MoAbs, human serum samples were used in each given experiment as reported in the Results section. Tested samples were then washed by adding 0.5 ml of washing buffer and then centrifuged for 5 minutes at 200*×g*. The supernatant was aspirated and the pellet re-suspended in 100 µl capture micro-bead diluent for serum and plasma (Becton Dickinson), then 50 µl of the detection diluent containing phycoerythrin- (PE; 5–20 µl) or fluorescein isothiocyanate-conjugated secondary antibody (FITC; 20 µl) were added to each tube, vortexed, and then incubated for 1 h at room temperature in the dark. Secondary antibodies used in our ABA assay were as follows: a FITC conjugated goat anti-rabbit IgG (GenWay Biotech Inc., San Diego, USA) was used for rabbit primary PoAbs (10 µl for each sample); MoAbs were detected using 5 µl of PE-conjugated goat anti-mouse (Dako Cytomation, Milan, Italy); human IgE were detected using 20 µl of PE-conjugated anti-human IgE (Becton Dickinson). The detection diluent was added to all secondary antibodies to reach the final volume of 50 µl. Samples were then washed once as described above and finally re-suspended in 300 µl of washing buffer.

Specimens were analyzed according with the CBA assay for cytokine detection [10], [11] by using the FACSAria flow cytometer. FACSDiva software (Becton Dickinson) was used for data capturing and a first analysis. Briefly, cytometer setup micro-beads adsorbed with FITC- or PE-positive control detector reagents were used to adjust the side scatter (SSC), the forward scatter (FSC), and the FL1, FL2, FL3, and FL4 photomultiplier tube settings so that each of the four micro-bead types clustered within the first decade of fluorescence. The analysis of a human negative control serum, obtained from our sera bank and defined negative on the basis of a negative allergy clinical history and repeated ISAC negative testing, was used to verify that photomultiplier tube settings and compensation were correct. Upon analysis of raw data, the median fluorescence intensity (MFI) of each micro-bead cluster was quantified.

Five tubes labelled A9, PE-F1, F1, F9 and A1 were prepared. After vortexing, 200 µl of washing buffer was added to each tube followed by 25 µl of the corresponding setup micro-beads. The following parameters were edited: FSC-A, FSC-W, SSC-A, SSC-W, PE-A, APC-A and APC-Cy7-Then, the following plots were created on a global worksheet : FSC-A/SSC-A dot plot, APC/APC-Cy7 dot plot and a PE histogram. The FSC-A and SSC-A were set to log and a statistic view was created showing the FSC-A and the SSC-A means. Measurements were performed counting at least 500 events for each analysis. The FSC and SSC thresholds were adjusted using the mean channel as a guideline. In the FSC-A *vs* SSC-A dot plot a region was created that includes the singlet population of micro-beads. The statistics view was edited to display the PE, FITC, APC and APC-Cy7 median fluorescence values. Through the singlet gate, the A9 setup micro-beads were run and the voltage of APC and APC-Cy7 were adjusted until obtaining a 160,000±2,000 mean for each parameter. Through the singlet gate the PE-F1 tube was run and the PE voltage was adjusted until a mean value of 65±5 was reached.

**Figure 1:**
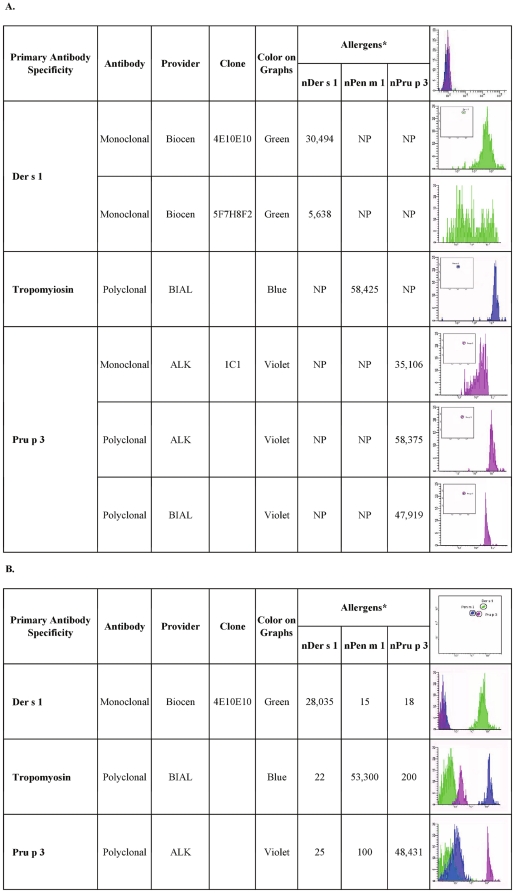
Detection of allergen conjugation on micro-beads using mouse monoclonal and rabbit polyclonal antibodies. Panel A: Each antibody preparation was tested on the respective allergen-coupled micro-bead. The other two micro-beads were not in the tube. NP: Not performed in this singlepex testing. * Values are expressed as Median Fluorescence Intensity [MFI]. Upper right corner: an example of blank fluorescence reading of the three conjugated micro-beads without serum. Panel B: Antibodies selected on the basis of the findings reported in panel A were tested on the three allergen-coupled micro-beads, all found in the same testing tube. * Values are expressed as Median Fluorescence Intensity [MFI]. Upper right corner: an example of micro-bead fluorescence scatter plot and micro-bead clusters.

**Figure 2:**
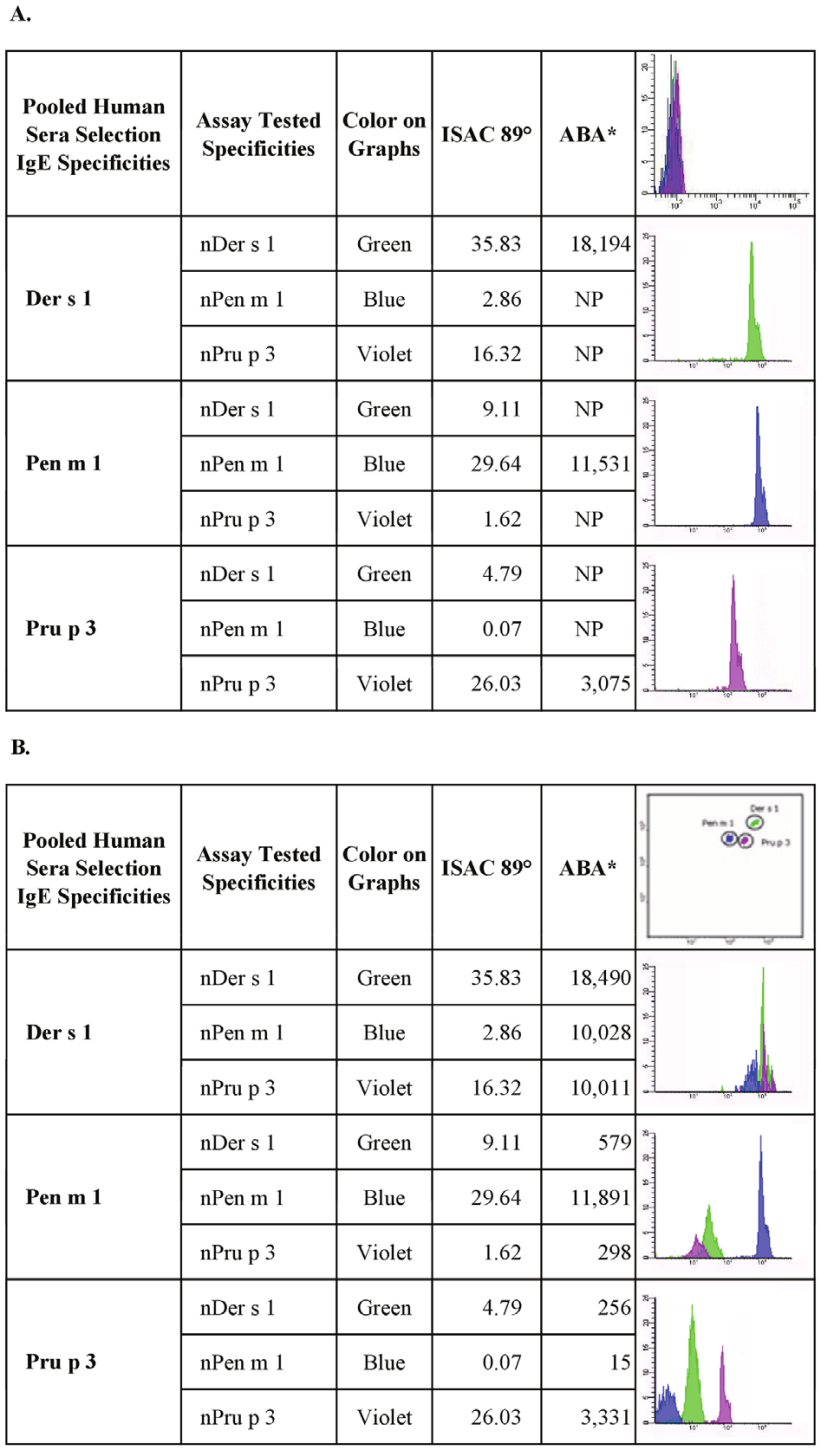
Detection of allergen conjugation on micro-beads using human pooled sera containing specific IgE. Selected pooled human sera with IgE specific for the three candidate allergens were tested to verify that allergen coupling was acceptable also for human immunoglobulin determination and to estimate interference among different allergen-conjugated micro-beads. Panel A: Each serum pool was tested on the respective allergen-coupled micro-bead. The other two micro-beads were not in the tube. NP: Not performed in this singlepex testing. ° Values are expressed as kU/l. * Values are expressed as Median Fluorescence Intensity [MFI]. Upper right corner: an example of blank fluorescence reading of conjugated micro-beads without serum. Panel B: Serum pools were tested on the three allergen-coupled micro-beads present in the same tube. ° Values are expressed as kU/l. * Values are expressed as Median Fluorescence Intensity [MFI]. Upper right corner: an example of micro-bead fluorescence clusters.

ABA-measured IgE values were expressed as MFI as given by the final readout of the cytometer. No standard curves, as reported in other studies [24], were created to normalize MFI and obtain ABA IgE semi-quantitative values as for the ISAC system. A blank reading for each run, made with conjugated micro-beads and micro-beads plus serum without secondary antibody to measure background fluorescence, was performed always with comparable very low range fluorescence results (data not shown). As a totally negative MFI was seldom recorded for allergic sera when the PE-anti-IgE was added (47 out of 1,519 determinations, 3.1%, Table S1), we tentatively set a negative cut off point for the new detection system at a value being the mean plus three standard deviations of the ABA IgE values of all the ISAC IgE negative tests used in the present study, plus all the conjugated micro-bead fluorescence values obtained before serum testing and evaluated at every ABA run (example given in Figures 1, panel A and Figure 2, panel A). Overall 799 ISAC IgE negative results and 238 blank micro-beads values were combined, resulting in a negative cut off value equal to 200 MFI. The cut off value was used for the IgE data analysis of the last set of 336 sera.

### Data management and Statistics

Demographic and clinical data were recorded for all patients at admission by using the InterAll software, a customized allergy electronic record for diagnostic and clinical data storing (version 3.0, Allergy Data Laboratories s.c., Latina, Italy). All laboratory data obtained in the present study along with all ISAC and ABA testing results were stored in the patients' personal records and analyzed.

Graphpad Prism version 5.02 was used for statistics and graphs (Graphpad Software Inc., La Jolla, CA, USA). The frequencies of events were compared by using the Fisher's exact test and the χ^2^ test was used for the two by two contingency tables. The non-parametric Spearman correlation coefficient (r) was used to compare the IgE values obtained by the two testing systems. All tests were used with two-sided options and the statistically significant level was set at a p value<0.05.

## Results

### Allergenic molecules conjugation evaluated using specific polyclonal and monoclonal antibodies

To begin validation of the ABA system, the proof of a successful allergen conjugation on the blank micro-beads was searched in three independent experiments. The recognition of nDer s 1, nPen m 1 and nPru p 3 by specific non-human antibodies was tested with both a singleplex and a multiplex approach. In the singleplex assays, every molecule coupled with the micro-beads was tested with its specific antibody. In the multiplex approach, the three molecules, coupled with their respective micro-beads, were tested simultaneously with each of the antibodies to check any reciprocal interference.

Two different MoAbs to nDer s 1 were tested; the 4E10/E10 showed that the allergen was specifically coupled with the micro-bead, with a higher performance when compared to the MoAb 5F7/H8F2 (Figure 1, panel A). nPen m 1 conjugation, tested using the tropomyosin specific PoAb, produced very good results showing a successful conjugation of the molecule (Figure 1, panel A). nPru p 3 conjugation to the micro-beads was evaluated by means of one MoAb and two different PoAbs in independent experiments. As shown in Figure 1, panel A, all the anti-Pru p 3 antibodies showed an overall good recognition of the conjugated nPru p 3, though the two PoAbs performed more homogeneously in terms of a narrower range of fluorescence emission when compared to the MoAb, anyway providing the proof of a proper conjugation of the molecule on the blank micro-bead.

In order to exclude a possible interference, the three different micro-beads conjugated with nDer s1, nPen m 1, nPru p 3, were mixed in the same tube to be tested. The best performing antibodies among those reported above were used, namely the anti-Der s 1 MoAb, clone 4E10/E10, the anti-tropomyosin and the anti-Pru p 3 PoAb supplied by Alk-Abelló. Figure 1, panel B, shows the selective recognition of the three allergens by the respective antibody in the multiplexing approach and the absence of interference among different allergen conjugated micro-beads. Reproducibility of the results obtained by testing the selected polyclonals anti-tropomyosin and anti-Pru p 3, and the monoclonal anti-Der s 1 was explored in three different experiments. All positive results were reproduced but a very high coefficient of variability was obtained (approx 50% considering all the experiments together; data not shown). The same antibodies were then used to create dilution curves in separate experiments. Graphs, as reported in Figure S1, show acceptable dilution curves, but also a great variability in the allergen/antibody performances in the ABA system by the three combined antigen/antibody preparations.

None of the control MoAbs or PoAbs produced any unspecific binding with the three allergen-conjugated beads, since the recorded fluorescence signal was always within the lowest background range (data not shown).

### Allergenic molecule conjugation and reactivity evaluated using specific pooled human IgE

To validate data obtained by non-human antibodies and obtain the first proof of feasibility of the new testing, IgE recognitions of the three molecules were analysed. Pooled sera from individual donors reactive to nDer s 1, nPen m 1 and nPru p 3 were used in three independent experiments. As for non-human antibodies, the reactivity profiles of the pooled sera were evaluated both in ABA singleplex and multiplex. Singleplex results, obtained by testing the single micro-bead with the specific serum pool, reflected the specific IgE recognition pattern obtained using the ISAC system and detailed in Figure 2, panel A. ABA IgE recognition patterns looked much alike those obtained by non-human antibody testing as reported above in Figure 1.

When the three micro-beads were tested together, they produced the following results (Figure 2, panel B): the Der s 1-selected pooled sera, known to have ISAC detected IgE to nPen m 1 and nPru p 3, showed specific IgE reactivity to all the molecules tested in the ABA multiplexed array. The nPen m 1-selected specific pooled sera gave just one strong signal on nPen m 1, but to a minor extent recognized also nDer s 1 and nPru p 3, comparably to results previously obtained using the ISAC test. In the case of the LTP-selected pooled sera, nPru p 3 produced a strong positive signal, whilst a slight IgE reactivity was observed for nDer s 1, and none to nPen m 1, in agreement with ISAC testing.

**Figure 3:**
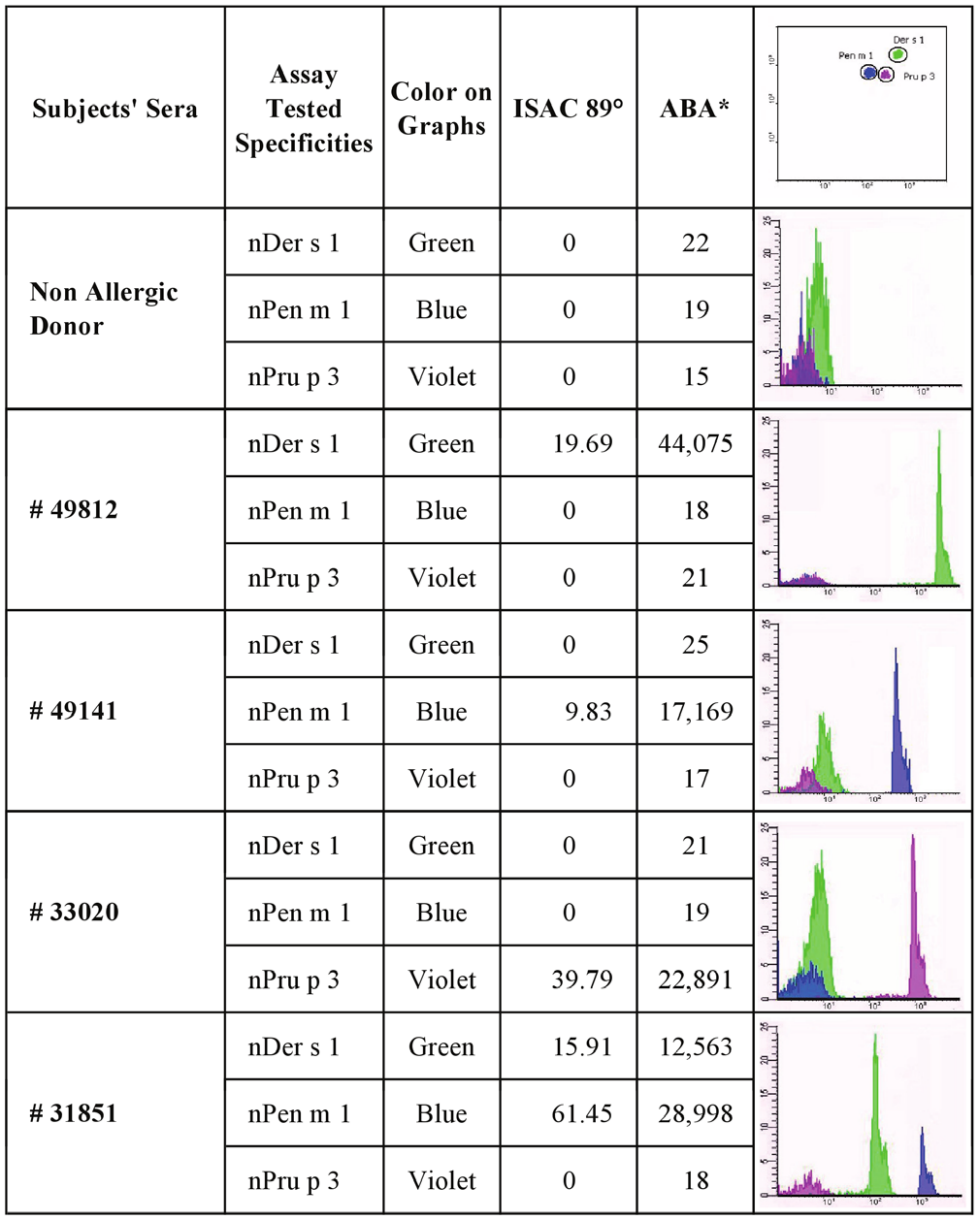
Testing human sera containing specific IgE on allergen conjugated micro-beads. Three human individual serum samples (49812, 49141, 33020), each of them known to have specific IgE to one allergen and not to the others were tested on the three micro-beads present in the same tube. Serum 31851 was from a subject recognizing nDer s 1 and nPen m 1, but not nPru p 3. A non allergic donor was used for control purposes. # Serum sample numbers; ° Values are expressed as kU/l; * Values are expressed as Median Fluorescence Intensity [MFI]. Upper right corner: an example of micro-bead fluorescence clusters.

Additional experiments according to this validation approach included the evaluation of three mono-reactive patients to either nDer s 1, or to nPen m 1, or to nPru p 3, and an additional individual having IgE to both nDer s 1 and nPen m 1. As shown in Figure 3, all the known reactivities were confirmed, thus indicating the specificity of IgE recognition using the ABA test in perfect agreement with ISAC IgE testing.

### ABA *versus* ISAC Comparative IgE testing

One hundred and thirty-seven allergic individuals were selected on the basis of their ISAC IgE reactivity to one of the three allergens of our study and not to the other two. Twenty-nine nDer s 1 positive patients (16 males, median age 13 years, range 2–39 years), 28 nPen m 1 positive (16 males, median age 14 years, range 3–60 years), and 55 nPru p 3 positive (25 males, median age 28 years, range 9–69 years) were selected. Twenty-five allergic subjects (18 male, median age 19, range 2–49 years old), without any of the three above positive IgE determinations on ISAC acted as negative controls. Without a set cut off value for the ABA IgE detection system, any value above the no fluorescence signal was considered as positive.

**Figure 4:**
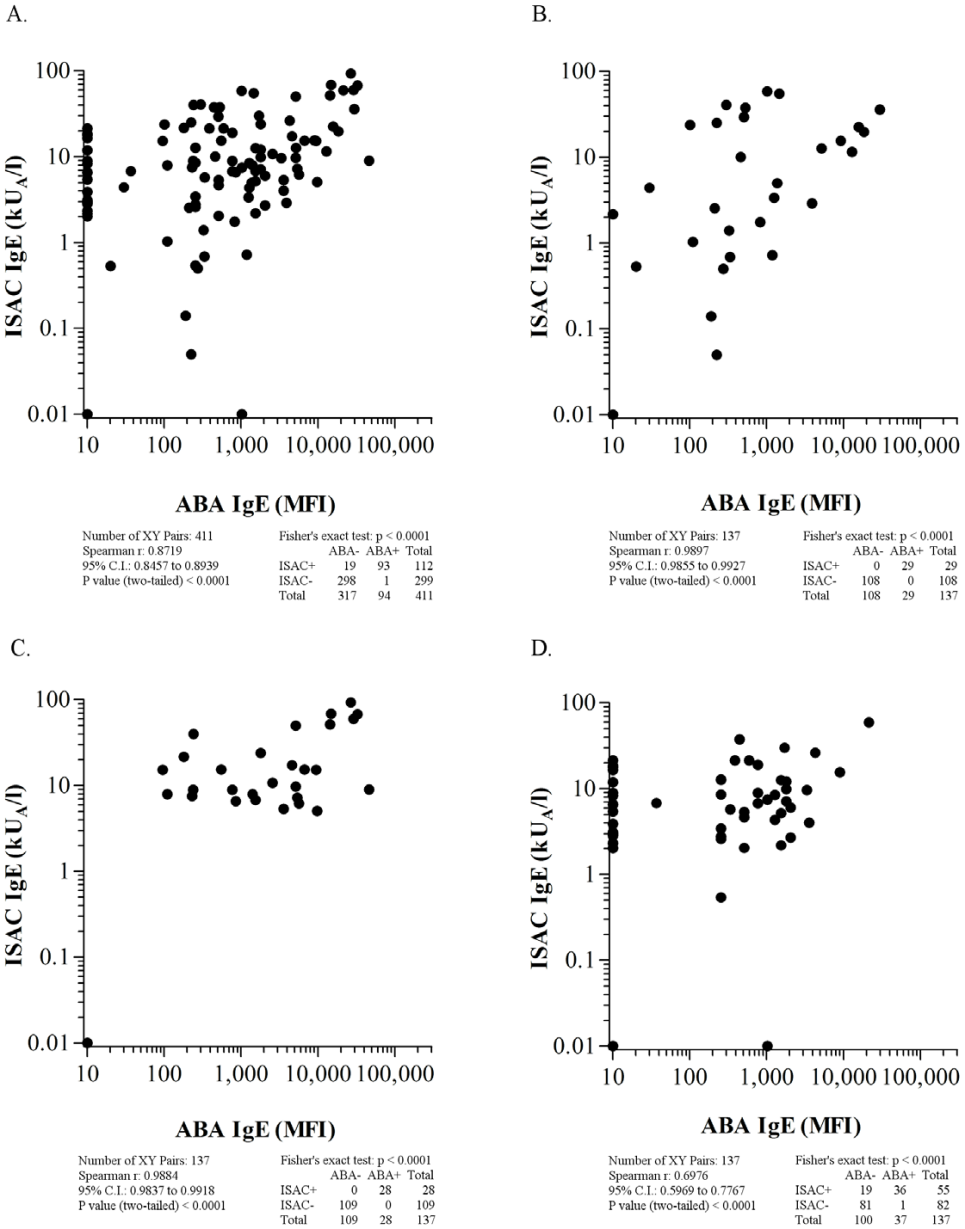
ABA *versus* ISAC correlation results on 137 serum samples selected on the basis of nDer s 1, nPen m 1, and nPru p 3 mutually exclusive IgE positivity are reported. Panel A: All 411 IgE values, obtained by testing the three allergens; Panel B: 137 IgE results obtained on nDer s 1 allergen; Panel C: 137 IgE results obtained on nPen m 1 allergen; Panel D: 137 IgE results obtained on nPru p 3 allergen. For graphical visualization needs on log scales, zero values for ABA were set at 10 MFI on the X axis, and at 0.01 kU/l for ISAC values on the Y axis. The Spearman r correlation coefficient, the χ^2^ and the Fisher's exact tests were used where applicable.

When comparing ABA IgE reactivity to the ISAC IgE testing in the 137 samples (411 determinations), a statistically significant high correlation was found (r = 0.87, CI 0.84–0.89; p<0.0001) (Figure 4, panel A), confirmed by the contingency table evaluation (p<0.0001). Results were anyhow scattered across a quite large area and some ISAC positive/ABA negative results were evident rather than the opposite. When a discrete allergen-driven analysis was performed, all samples tested for nDer s 1 (Figure 4, panel B) and nPen m 1 (Figure 4, panel C) were ABA IgE positive, with very high r values, equal to 0.98 (CI 0.92–0.99; p<0.0001) and 0.988 (CI 0.98–0.99; p<0.0001), respectively. When comparing nPru p 3 reactivity (Figure 4, panel D), 36 patients were ISAC/ABA double positive, whilst 19 turned out to be negative after ABA test, but ISAC positive, and 1 case was ABA positive, but lacking ISAC IgE reactivity. Statistics were as follows: r = 0.7 (C.I. 0.60–0.78), Fisher's exact test p<0.001. All 25 control allergic subjects not sensitized to nDer s 1, nPen m 1 and nPru p 3 were negative for ABA IgE testing on the three candidate molecules.

### ABA multiplex IgE testing on additional allergenic molecules

**Figure 5:**
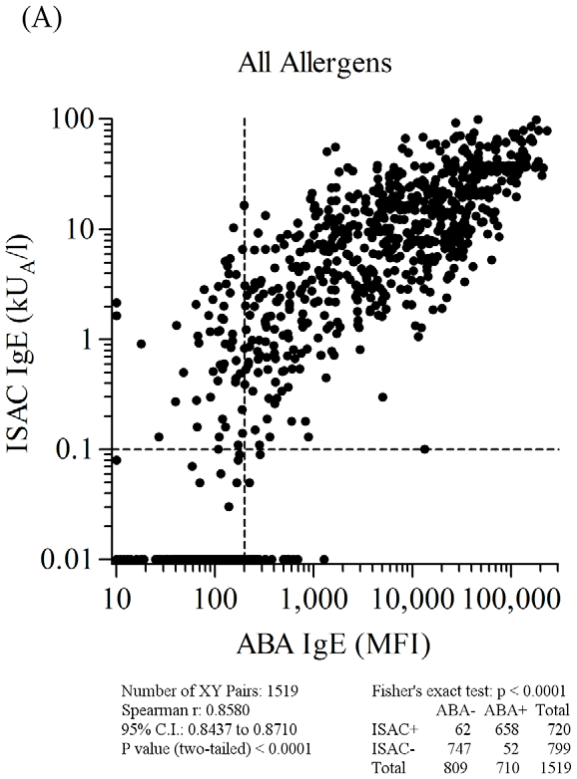
ABA *versus* ISAC IgE correlation results on 1,519 serum samples selected on the basis of any of the allergen specificities reported in Table S1, using the two micro systems. Letter flag (A) in figure 5 indicates it as part of the results shown also in figures 6, 7, and 8. Consecutive letters are used on purpose to recall result type continuity across the four figures. Allergen nature, being either natural or recombinant, matched for both tests. Vertical dashed lines represent the arbitrary ABA negative cut off value. Horizontal dashed lines mark the value range where ISAC IgE determinations are not always reproducible (unpublished data). For graphical visualization needs on log scales, zero values for ABA were set at 10 MFI on the X axis, and at 0.01 kU/l for ISAC values on the Y axis. The Spearman r correlation coefficient was calculated and the χ^2^ test was used for statistical purposes. Statistical results are reported below the graph.

**Figure 6:**
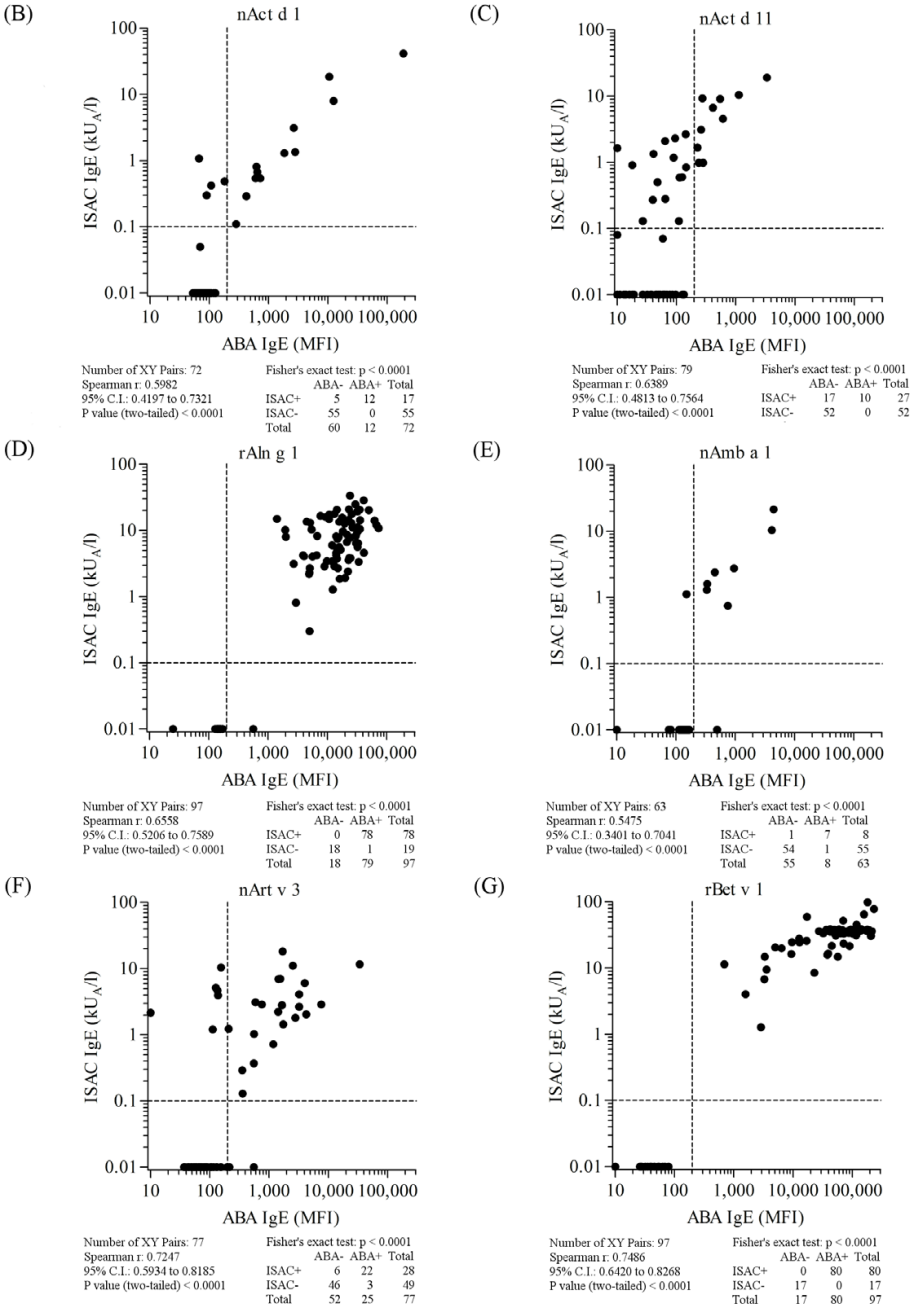
ABA *versus* ISAC correlation results on serum samples selected on the basis of the allergen specificities reported in each panel and listed in Table S1. Letter flags, namely B, C, D, E, F, G, in figure 6 indicate them as parts of the results shown also in figures 5, 7, and 8. Consecutive letters are used on purpose to recall result type continuity across the four figures. Allergen nature, being either natural or recombinant, matched for both tests. Vertical dashed lines represent the arbitrary ABA negative cut off value. Horizontal dashed lines mark the value range where ISAC IgE determinations are not always reproducible (unpublished data). For graphical visualization needs on log scales, zero value for ABA was set at 10 MFI on the X axis, and at 0.01 kU/l for ISAC value on the Y axis. The Spearman r correlation coefficient was calculated and the Fisher's exact test was used for statistical purposes. Statistical results are reported below each graph.

**Figure 7:**
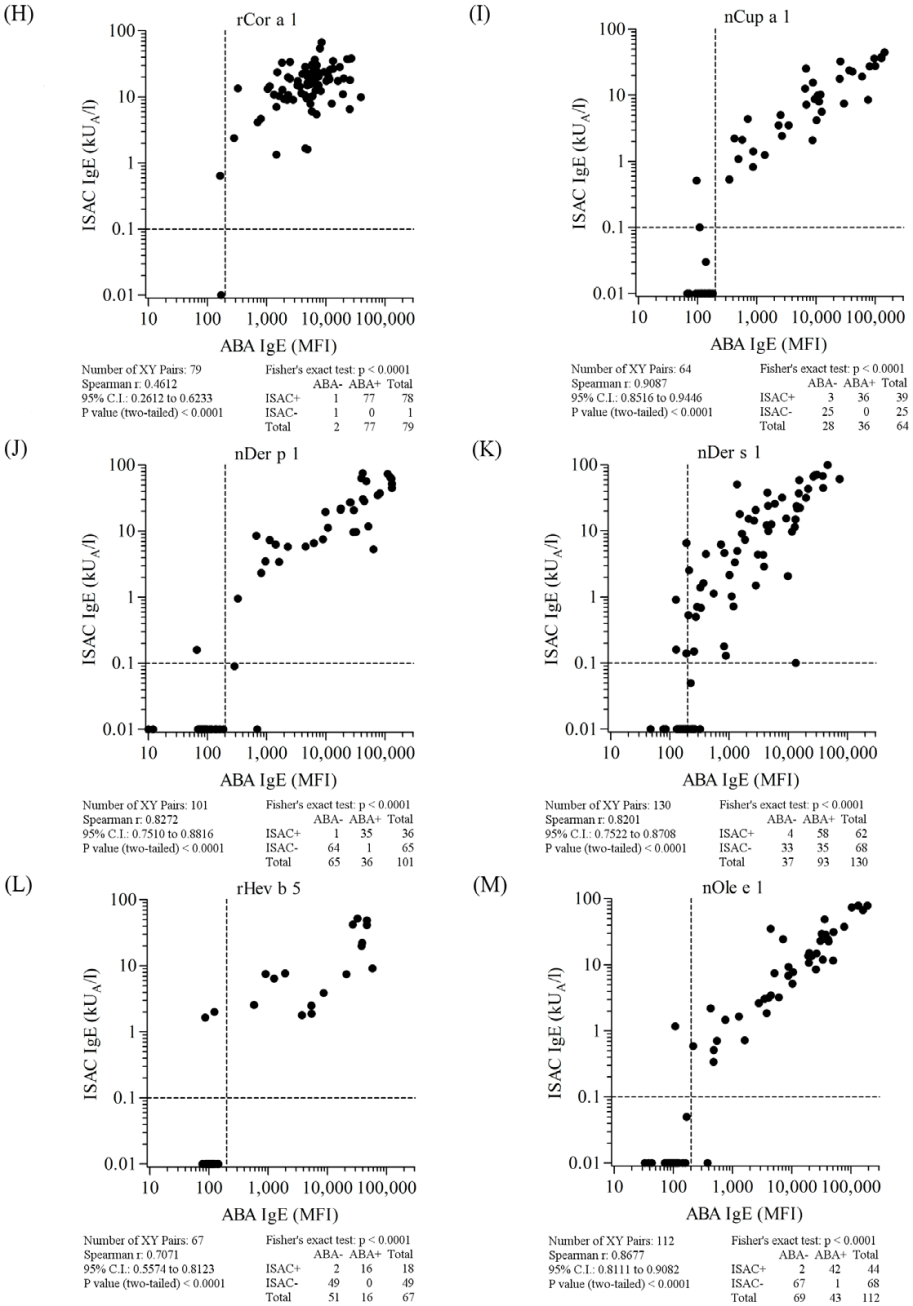
ABA *versus* ISAC correlation results on serum samples selected on the basis of the allergen specificities reported in each panel and listed in Table S1. Letter flags, namely H, I, J, K, L, M, in figure 7 indicate them as parts of the results shown also in figures 5, 6, and 8. Consecutive letters are used on purpose to recall result type continuity across the four figures. Allergen nature, being either natural or recombinant, matched for both tests. Vertical dashed lines represent the arbitrary ABA negative cut off value. Horizontal dashed lines mark the value range where ISAC IgE determinations are not always reproducible (unpublished data). For graphical visualization needs on log scales, zero value for ABA was set at 10 MFI on the X axis, and at 0.01 kU/l for ISAC value on the Y axis. The Spearman r correlation coefficient was calculated and the Fisher's exact test was used for statistical purposes. Statistical results are reported below each graph.

**Figure 8:**
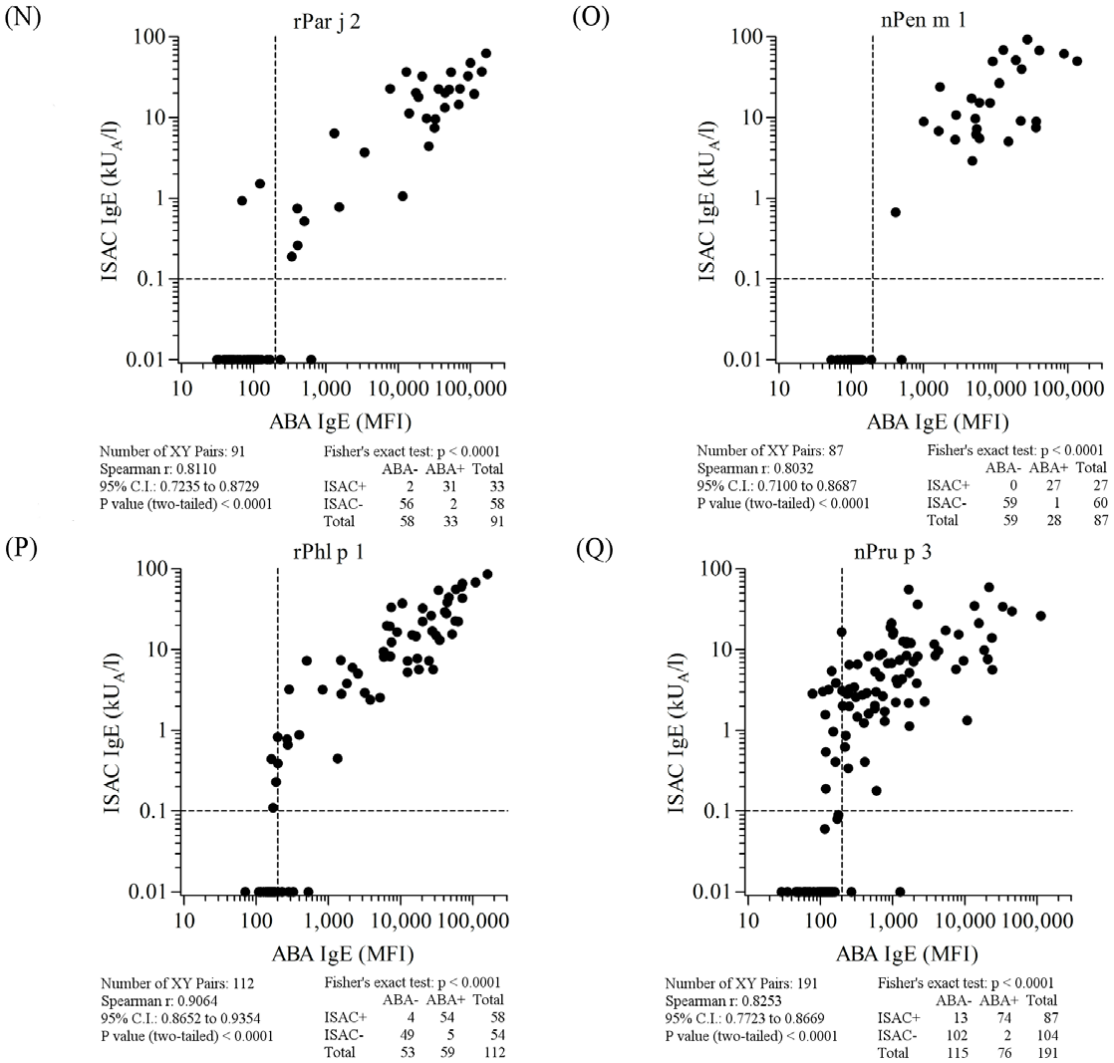
ABA *versus* ISAC correlation results on serum samples selected on the basis of the allergen specificities reported in each panel and listed in Table S1. Letter flags, namely N, O, P, Q, in figure 8 indicate them as parts of the results shown also in figures 5, 6, and 7. Consecutive letters are used on purpose to recall result type continuity across the four figures. Allergen nature, being either natural or recombinant, matched for both tests. Vertical dashed lines represent the arbitrary ABA negative cut off value. Horizontal dashed lines mark the value range where ISAC IgE determinations are not always reproducible (unpublished data). For graphical visualization needs on log scales, zero value for ABA was set at 10 MFI on the X axis, and at 0.01 kU/l for ISAC value on the Y axis. The Spearman r correlation coefficient was calculated and the Fisher's exact test was used for statistical purposes. Statistical results are reported below each graph.

To show the feasibility of the system as a flexible multiplex array and to explore if we introduced any bias in previous experiments due to the limited number of sera and allergens, we additionally tested 336 sera from allergic patients, 171 females (50.9%), median age 27 years, range 2–80 years, 70 of them were younger than 12 years old, 17 in pre-school age. Full demographics are reported in Table S1. IgE were assessed using 16 allergenic molecules, namely nAct d 1, nAct d 11, rAln g 1, nAmb a 1, nArt v 3, rBet v 1, rCor a 1, nCup a 1, nDer p 1, nDer s 1, rHev b 5, nOle e 1, rPar j 2, nPen m 1, rPhl p 1, and nPru p 3. Results for each allergen are reported in Table S1 and Figure 5, 6, 7, 8, as indicated in the legends. Each sample was selected on the basis of the ISAC IgE positive results to one of the above allergen specificities, but other positive and negative ISAC IgE results were considered and tested as well. Before each ABA IgE testing session selected allergen micro-beads bearing the leading specificity of each sample included in our study were mixed in all tubes. Thus a different set of allergens was used in each session, always including between 1 and 11 different allergens, as detailed in Table S1.

When comparing all ABA/ISAC results obtained after a total of 1,519 unique determinations on each system, a high correlation of measured IgE values (r = 0.86, C.I. 0.84–0.87; p<0.0001) and a very high agreement of positive and negative tests (χ^2^ 1096.1, p<0.0001) were found (Figure 5). Discrete allergen specificity was then analyzed to see whether different behaviors of the ABA IgE testing system could be related to the different molecules used for the test. All graphical representations and statistical evaluations are exhibited for each allergen in the respective panels in Figures 5, 6, 7 and 8, as detailed by the letters in the Figure legends. Consecutive letter assignment to each allergen panel was made on purpose across the four Figures to recall result type continuity.

The number of single observations per allergen ranged between n = 63 (nAmb a 1, Figure 6, panel E and Table S1) and n = 191 (nPru p 3, Figure 8, panel Q and Table S1). Correlation values were all statistically significant at the same level (p<0.0001) with the Spearman r coefficient ranging between 0.46 (rCor a 1, Figure 7, panel H) and 0.91 (nCup a 1, Figure 7, panel I). The best performance rates, considering the concordance of positive and negative tests, were recorded for rAln g 1, nAmb a 1, rBet v 1, rCor a 1, nDer p 1, rHev b 5, rPar j 2, and nPen m 1 allergens. The allergen where the highest ABA/ISAC discrepancies were recorded was nDer s 1 (Figure 7, panel K), whereas rBet v 1 (Figure 6, panel G) had no discrepancies.

The nDer s 1 ABA IgE results (Figure 7, panel K) quite often showed to be slightly above the in-house defined cut off value compared to the negative value obtained with the same preparation immobilized on the ISAC microarray, whereas the opposite discrepancy was recorded again for the nPru p 3 (Figure 8, panel Q), with 13 positive IgE values on the ISAC system not replicated by the ABA assay. It is important to note that the same nDer s 1 preparation was used in the two testing system, whereas the two preparations on ABA and ISAC were from different providers.

To evaluate the interference of high total IgE values on the ABA IgE detection system, 117 samples having estimated total IgE values were selected (Table S1). Two subsets were created where the total IgE value was either below or above 1,000 IU/l. The first subset of 54 samples had total IgE values within the range of 4–924 IU/l, whereas 63 samples in the second subset had total IgE within the range of 1,087–39,038 IU/l. The number of positive ABA IgE tests (>200 MFI), when the ISAC IgE testing was negative, it was compared between the two subsets, taking into account the number of negative ISAC tests in the low and high subgroups, equal to 126 and 351, respectively. Twelve samples (9.52%) in the subset with total IgE<1,000 IU/l had ABA IgE values above the cut off, whereas 35 (9.97%) of the high/very high total IgE samples were above the ABA cut off value. The two frequencies were not statically different when evaluated with the Fisher's exact test.

**Figure 9:**
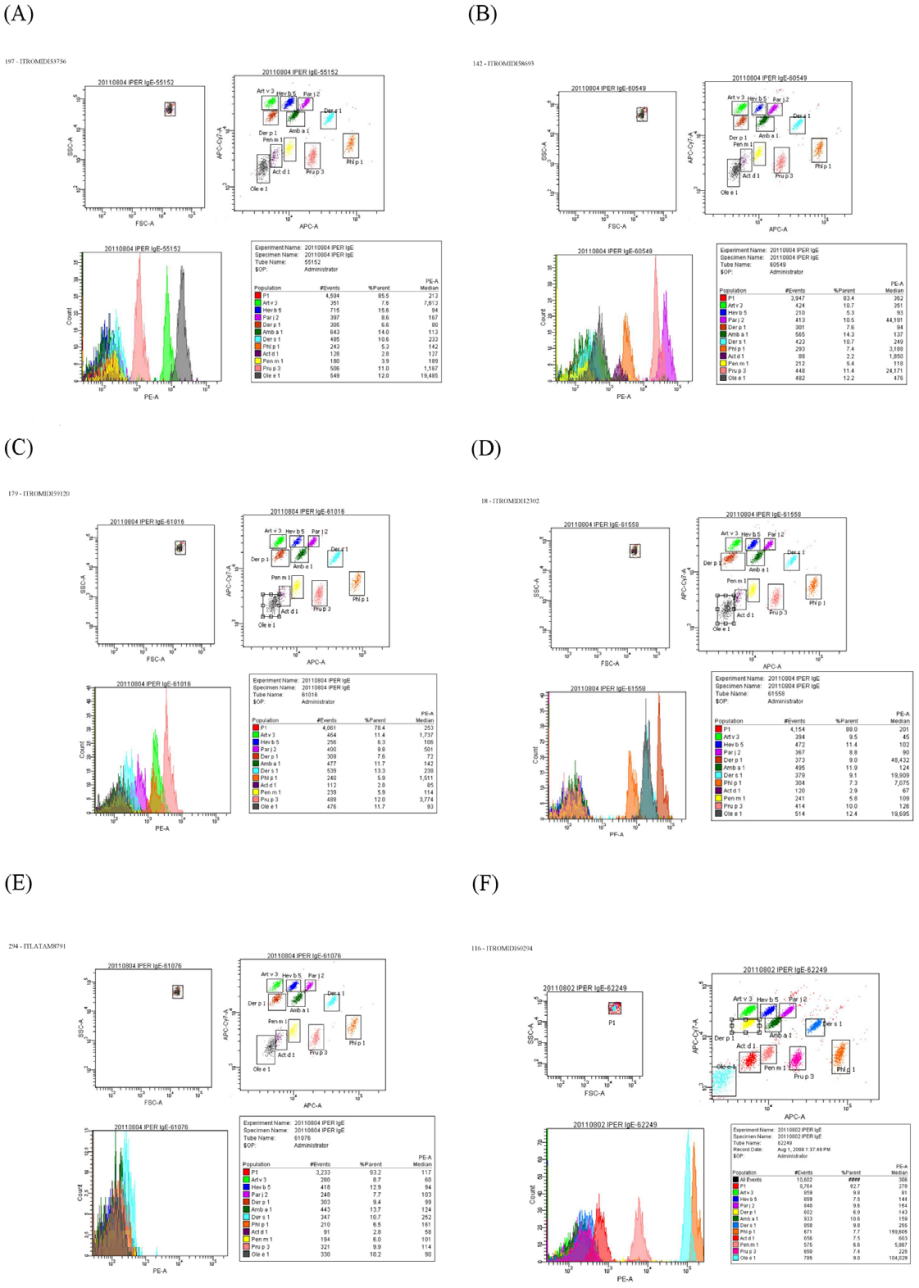
Selected examples of Allergen micro-Beads Array (ABA) IgE testing report. Eleven different micro-beads were tested in the same tube in each run for each sample. Examples were selected on purpose among the samples with high total IgE. All IgE positive and negative ABA results matched the ISAC results. Numbers in the upper left corner indicate the Table S1 row numbers and patients' ID. In each panel: the upper left graph shows clustered micro-beads by their dimension; the upper right: scatter plots of each fluorescent bead; the lower left: fluorescence intensity and event counts; the lower right: summary table with median fluorescence values. Samples reported in each of the six panels had the following total IgE values: Panel A = 9,730 IU/l; Panel B = 1,351 IU/l; Panel C = 1,220 IU/l; Panel D = 1,931 IU/l; Panel E = 20,900 IU/l; Panel F = 23,540 IU/l.

Overall, Table S1 fully reports on single patient ABA IgE testing data and recorded profiles, showing the configurable aspect of the new assay. In order to show how a real ABA testing report from the cytometer looked like, six examples of ABA IgE testing were selected among those displayed in Table S1 (marked in blue) and shown in Figure 9. Eleven different micro-beads were tested in the same tube in each run for each sample. Examples were selected on purpose among the samples with high total IgE, values ranging between 1,220 and 23,540 IU/l. All IgE positive and negative ABA results matched the ISAC results. Reports clearly showed how the scatter plot of each micro-bead discriminated the used ones by their individual fluorescence, how the micro-bead count and fluorescence intensity were displayed and measured, and the calculation to obtain the median fluorescence value of each tested allergen.

To evaluate the reproducibility of the IgE determinations performed with the ABA system, we selected 114 consecutive samples from subjects as in the Table S1, obtaining 283 paired determinations, and all performed in two separated ABA runs. The experiments used 15 of 16 allergens under study. Not all allergens and subjects were tested the same number of time and aggregated data are provided in terms of IgE value correlation and concordance. Supplementary Figure S2 shows the correlation graph and the statistical evaluations are reported in its legend. Undoubtedly good results were obtained and the lack of a reference curve seems not to affect the IgE detection between the two distant runs at least in terms of positive/negative concordance. The experiment could be of course be biased by the non random selection of samples which has been performed on the basis of serum sample availability.

## Discussion

In the present study we report for the first time the feasibility of a new multiplex system customized for detecting specific IgE testing in serum, which uses allergenic molecules coupled with micro-beads undergoing fluorescence measurement by the cytometer available in our lab facilities. Starting from the coupling of the allergens with the micro-beads to the overall extensive IgE testing results, the aims of our study were fulfilled. Major achievements were the almost overlapping results obtained when comparing ABA to the control system and the suitability of the ABA IgE testing system for all the allergenic molecules tested so far. The cytometric ABA system herein described was not affected by background noise frequently observed in other serum-based immunoassays. This was clearly shown in several of our experiments, mostly in the one using samples with very high total IgE values. Furthermore, since we have not set a system negative cut off using a reference curve, our arbitrary cut off worked almost perfectly, with the exception of nDer s 1 where positive values were quite often slightly above the cut off unless the comparative ISAC IgE testing was negative. Since this behavior was not influenced by the testing of hyper-IgE samples, we considered it useful to evaluate again the quality of the preparation as it seemed to have some critical points when used on the ABA assay rather than on the ISAC microarray. Anyhow, creating a reference standard curve, by using one of the currently available approaches, will be a further step to qualify the ABA IgE testing system. The results obtained in reproducibility experiment further reinforce the idea that the ABA is a new suitable method to be used for IgE detection.

Data analysis showed a very good linear correlation of IgE detected by the flexible ABA system and the static ISAC microarray, with good to excellent r values, though some allergens showed a peculiar behavior. For instance, nPru p 3 tested on ISAC showed 11 clear-cut positive results compared to the negatives obtained by ABA testing. It is noteworthy that the two systems bear two different nPru p 3 preparations. An explanation for the discrepancy could be the different quality of purified molecules. In this regard, the use of the same preparation, as in the study by King et al. [16] to validate a new micro testing system, would reduce the number of variables to be evaluated. In fact, they developed a flexible multiplexed immunoassay for the detection of specific IgE using the XMap technology (Luminex Corporation, Austin, TX), which is based on a similar micro-bead system but needing a proprietary laser scanner for the final fluorescence estimation [16]. In the study, IgE results obtained using six allergens coupled on the microspheres, namely nDer p 1, nDer p 2, rBet v 1, nFel d 1, nCan f 1, and rPhl p 5, were compared to those obtained by testing using commercial ELISA plates (all six allergens) and using the streptavidin-CAP (Phadia) testing (Der p 1 and Der p 2). The latter is the most common singleplex testing system used worldwide for routine IgE measurements. For the purpose of the study, “flexibility” of the system was obtained by coupling the allergen on the ImmunoCAP solid phase using the biotin-streptavidin methodology [25]. This approach allowed the authors to use the same allergen preparations on the three testing systems. Very good results were obtained although on few allergens. Our results were almost comparable to those obtained by Erwin et al. for almost all allergens, but we were forced to not use the ImmunoCAP as a comparison, because not all molecules we studied had a commercially available ImmunoCAP preparation, and the use of a customized streptavidin-CAP or an ELISA assay would not be affordable in terms of allergenic molecule preparation amounts and costs, whether produced by our lab or purchased from commercial providers. Up to now no other reports have been found in literature using the same system for IgE detection, notwithstanding King et al. clearly showed the possibility of using microtechnology and allergenic molecules for IgE testing. As any other micro system - including our new ABA test - the main feature of the latter is that it needs very small amounts of any reagent. This holds true also when comparing the micro-bead-based system with a customizable system like the streptavidin-CAP, where larger amounts of allergen preparations and reagents are required [25]. The alternative use of the ImmunoCAP system based on currently available allergenic extracts was not feasible as well, as any of its extract is composed by known and unknown allergens resulting in a complex IgE measurement, which could mislead the conclusions of our study.

A different kind of microtechnology for IgE testing, first reported by Hiller and co-workers [6], is currently used worldwide and many studies have been published based on its use [26]. The ISAC test is based on allergenic molecules immobilized in a fixed number and kind on an activated glass slide [9]. This was basically the robustness of the study we recently published, where 23,077 subjects from the routine setting were all tested using the same panel of 75 molecules. The current version of the ISAC test (PMD) bears 103 natural and recombinant molecules. The ISAC testing system has been used in our lab for five years now and its routine use, inclusive of its “rigid” structure, allowed us to generate a wealth of research data comparing intervention cohorts and control cohorts, all within routine testing without much additional effort and resources [7], [20], [24], [27], [28]. Since this aspect can be envisaged as useful for the routine diagnostic workup and a combined well-structured epidemiological survey of different populations, the need of having the same advantages of microtechnology combined with a greater flexibility of the system was felt, for instance, when enrolling infants and newborns for studying limited aspects of their IgE reactivity, or when performing studies on allergens not yet available on the ISAC microarray or in any other system [18]. Thus the flexibility of the new ABA system could represent a major advantage if it allows choosing a personalized panel of allergens to test. As said, allergen-specific IgE measurement carried out simultaneously in a single run using the FACS facilities increases the chance of having a configurable microarray system for IgE measurements in department laboratories. Besides the flexibility in the panel of molecules to be used, the small amount of serum needed for the ABA test (50 µl), whatever the number of micro-beads/allergens to be tested, represents another key feature, as its use is possible when only scant amounts of biological fluids are available, such as in pediatric patient evaluation.

Compared to the ISAC microarray, the new ABA IgE assay has a limitation in the number of micro-beads that can be theoretically conjugated and analyzed at once. Thirty different micro-beads are currently available being quite far from the numbers of the “rigid” ISAC microarray, which have evolved from the early 74 to the current 103 immobilized allergens, without apparent expansion limitations. The practical aspect of ABA assay limitations is related to the number of combinations that should be obtained to have a larger number of different fluorescence on each micro-bead, and the problematic discrimination of micro-beads having the fluorescence cluster too close to one another. This aspect intuitively emerged from the ABA IgE testing reports shown in Figure 9, where the emission fluorescence proximity of each micro-bead avoided them from overlapping because we purposely used micro-beads that were not too close one another. The use of the nearest micro-bead led to an overlapping of the two closest bead fluorescence emissions. These technical limitations could hinder the expansion of the ABA system allowing a broad IgE testing for routine allergy diagnosis. Nevertheless, as shown in Table S1, the IgE co-recognition of nAct d 11, rAln g 1, rBet v 1, rCor a 1, structurally related allergens belonging to the same Bet v 1-like family, and, with more limited evidence, by nArt v 3 and nPru p 3, both belonging to the LTP family, suggests that the ABA assay is useful for studying IgE cluster reactivity on homologous molecules, as recently reported by Scala et al. [7] and described in more in detail in a recently accepted manuscript [29]. Moreover, testing allergens from species very close to one another, as in the case of nDer p 1 and nDer s 1, allows the comparative evaluation of the preparations, addressing the need of increasing the quality to improve performance in the diagnostic test.

Since we used mouse monoclonal and rabbit polyclonal antibodies in our study, employment of the ABA system to detect different human and non-human immunoglobulin isotypes in response to different allergens/antigens can be foreseen, thus facilitating larger experimental outcomes, mostly when using small-sized experimental animals.

In conclusion, we report the proof-of-concept that a quite simple flow cytometry micro-bead-based multiplex antigen array technology is suitable for the simultaneous detection of allergen-specific human IgE in not highly specialized laboratories with FACS facilities.

## Supporting Information

Figure S1 **Allergen micro-Bead Array (ABA) dilution curves of selected non-human sera.** All dilution curves started with the undiluted antibody preparation as provided by the manufacturer. Panel A: nDer s 1 and monoclonal antibody from Biocen 4E10E10; 1∶10 dilution factor; Panel B: nPen m 1 and the polyclonal antibody from BIAL; 1∶2 dilution factor; Panel C: nPen m 1 and the polyclonal antibody from ALK-Abelló; 1∶10 dilution factor.
(TIF)

Figure S2 **Allergen micro-Bead Array (ABA) reproducibility experiment on non-randomized sera.** Sera have been selected among those reported in Table S1 and on the basis of their availability for a further testing. 283 IgE determinations have been performed on sera from 114 subjects and 15 allergens but Act d 1 have been used. For graphical visualization needs on log scales, zero values for ABA were set at 10 MFI on both axes. The Spearman r correlation coefficient was equal to 0.91 (95% CI 0.88–0.93; p<0.001). The Fisher's exact test for contingency data table gave a concordance p value<0.0001 as only 17 discrepant results were recorded.
(TIF)

Table S1 **Allergen micro-Bead Array (ABA) for IgE detection in human sera. Aggregated and raw patient IgE data showing the flexibility of the system.** The 16 tested allergens are listed and hyperlinks are available for each of them cross-referencing to www.allergome.org. Panel A: Summary of the Table S1 patients' demographics and ABA IgE results. a: All tests performed for the given allergen (first row), positive tests (second row), median test value (third row); b: Positive tests (MFI>200; green background cells): lowest value (first row), highest value (second row), median value (third row); c: Negative tests (MFI<200; grey background cells): lowest value (first row), highest value (second row), median value (third row). Panel B: Raw IgE values for every single subject ranked by age are expressed as Median Fluorescence Intensity [MFI]. Green and grey cell background meaning throughout the table is as in Panel A sections (b) and (c). Selected examples of ABA IgE testing reports are shown in Figure 9. Subjects ID are marked with a blue background in this Table.
(XLS)
