# Supplementary figures and images for: Allergen Micro-Bead Array for IgE Detection: A Feasibility Study Using Allergenic Molecules Tested on a Flexible Multiplex Flow Cytometric Immunoassay
Source: PLoS One. 2012 Apr 17;7(4):e35697. doi: 10.1371/journal.pone.0035697 (PMC3328437; doi:10.1371/journal.pone.0035697)

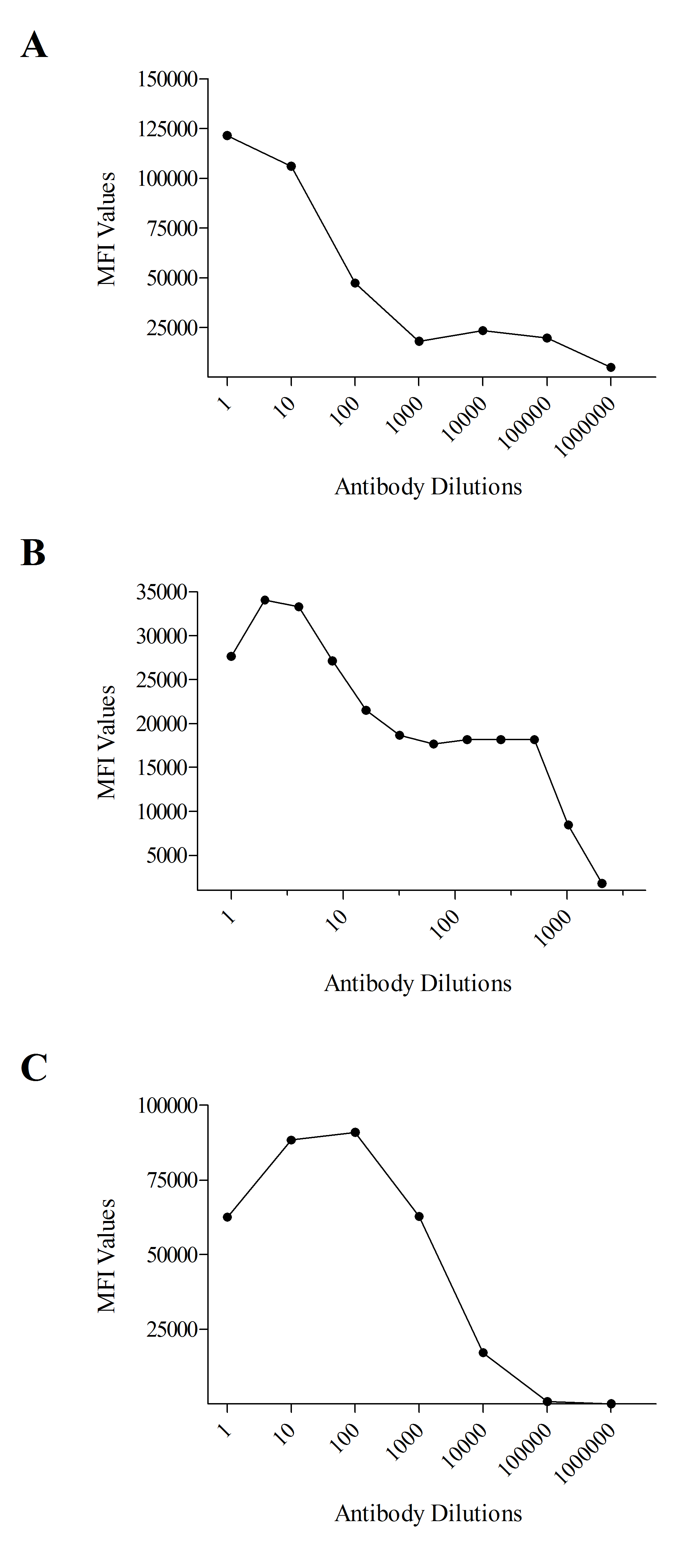

Supplement: Figure S1 — Allergen micro-Bead Array (ABA) dilution curves of selected non-human sera. All dilution curves started with the undiluted antibody preparation as provided by the manufacturer. Panel A: nDer s 1 and monoclonal antibody from Biocen 4E10E10; 1∶10 dilution factor; Panel B: nPen m 1 and the polyclonal antibody from BIAL; 1∶2 dilution factor; Panel C: nPen m 1 and the polyclonal antibody from ALK-Abelló; 1∶10 dilution factor. (TIF) [file pone.0035697.s001.tif]

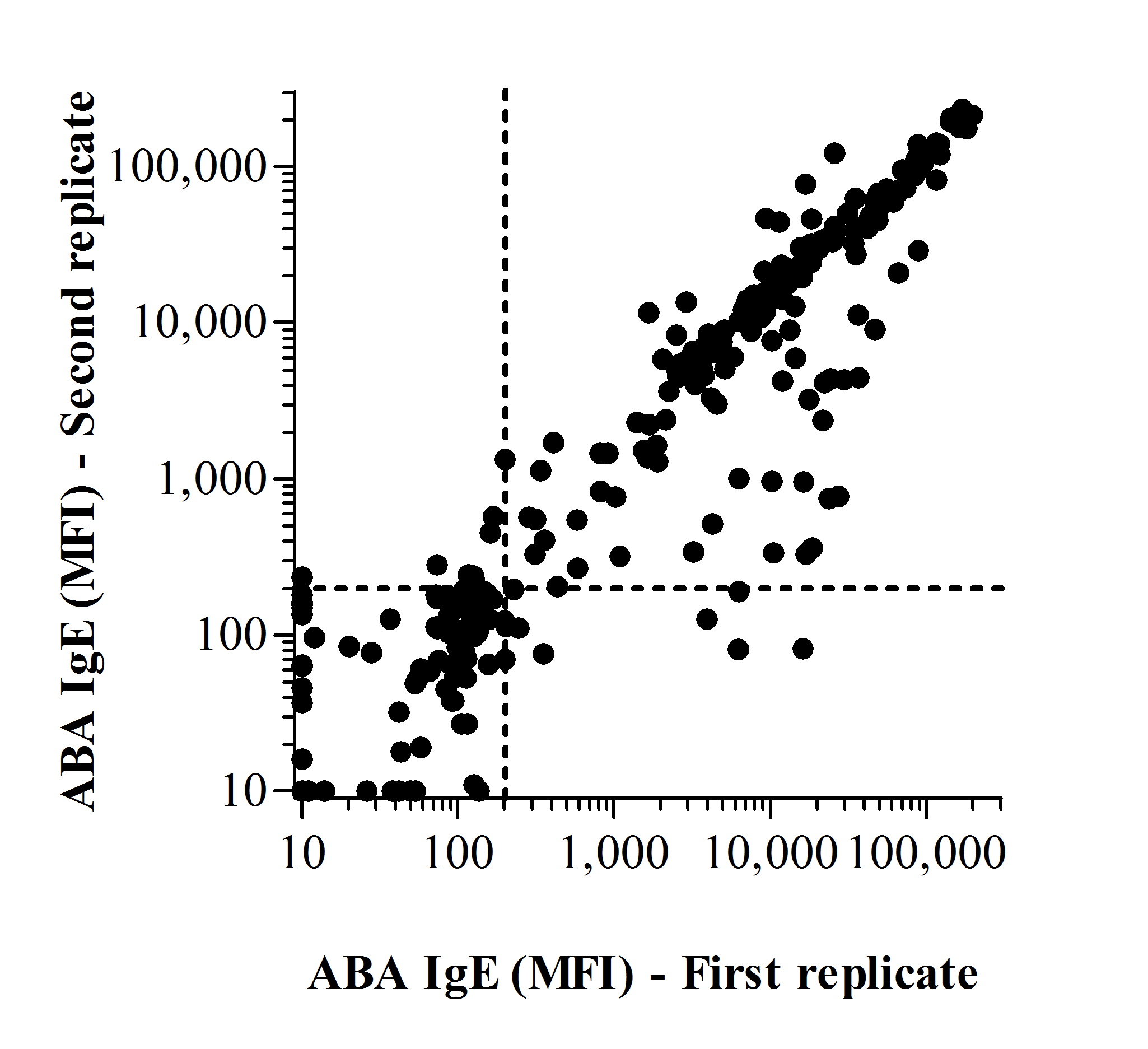

Supplement: Figure S2 — Allergen micro-Bead Array (ABA) reproducibility experiment on non-randomized sera. Sera have been selected among those reported in Table S1 and on the basis of their availability for a further testing. 283 IgE determinations have been performed on sera from 114 subjects and 15 allergens but Act d 1 have been used. For graphical visualization needs on log scales, zero values for ABA were set at 10 MFI on both axes. The Spearman r correlation coefficient was equal to 0.91 (95% CI 0.88–0.93; p<0.001). The Fisher's exact test for contingency data table gave a concordance p value<0.0001 as only 17 discrepant results were recorded. (TIF) [file pone.0035697.s002.tif]
